# Structured exploration of clinical trials data - finding the middle way

**DOI:** 10.1186/1745-6215-16-S2-P152

**Published:** 2015-11-16

**Authors:** James Weatherall

**Affiliations:** 1 AstraZeneca, Alderley Park, Cheshire, UK

## 

The traditional statistical analysis of data from clinical trials tends to follow a conservative approach centred around pre-specification, hypothesis testing and regulatory considerations. On the other hand, attempts to take a more exploratory approach are often criticised for being open-ended ‘data dredging’ exercises, lacking pre-specification, and failing to adequately control type I error.

If it is possible to find a ‘middle way’ between these two extremes, we can uncover invaluable additional insights about medicines - whether in development or approved. At AstraZeneca, our Structured Exploration capability was created to do just that. Examining patient-level clinical trials data more thoroughly can: (1) refine the target population for a medicine; (2) enhance our understanding of benefit-risk; (3) reignite the development of a compound otherwise shelved as having insufficient benefit in the overall trial population.

Our implementation of Structured Exploration centers around the selection of two specific data mining methods - Virtual Twins [1] and Inside-Out [2]. Virtual Twins has roots in the field of causal inference and counterfactuals, and provides a flexible framework within which to explore predictive subgroups. Inside-out turns the usual model formulation around, and instead of estimating the treatment effect on each adverse event separately, quantifies the ability of all the adverse events to classify patients to treatment.
